# Elderly mice with history of acetaminophen intoxication display worsened cognitive impairment and persistent elevation of astrocyte and microglia burden

**DOI:** 10.1038/s41598-024-65185-z

**Published:** 2024-06-20

**Authors:** Celso S. G. Catumbela, Rodrigo Morales

**Affiliations:** 1 Department of Neurology, The University of Texas Health Science Center at Houston, Houston, TX 77030 USA; 2 Centro Integrativo de Biologia y Quimica Aplicada (CIBQA), Universidad Bernardo O’Higgins, Santiago, Chile

**Keywords:** Acetaminophen (APAP), Acute liver failure (ALF), Elderly, Cognitive decline, Astrocytes, Microglia, Biochemistry, Neuroscience

## Abstract

Acetaminophen (APAP) is a leading cause of acute liver failure. The effect of APAP metabolite’s effects in the periphery are well characterized; however, associated consequences in the brain remain poorly understood. Animal studies on this subject are few and reveal that frequent APAP intake can trigger cerebral abnormalities that vary depending on the subject’s age. Alarmingly, experimental efforts have yet to examine associated consequences in elderly hosts, who correspond to the highest risk of medication overload, impaired drug clearance, and cognitive deficits. Here, we interrogated the cerebral and peripheral pathology of elderly mice submitted to monthly episodes of APAP intoxication since a young adult age. We found that weeks after the final episode of recurrent APAP exposure, mice exhibited worsened non-spatial memory deficit whereas spatial memory performance was unaltered. Interestingly, one month after the period of APAP intoxication, these mice showed increased glial burden without associated drivers, namely, blood–brain barrier disruption, cholesterol accumulation, and elevation of inflammatory molecules in the brain and/or periphery. Our experimental study reveals how recurrent APAP exposure affects the cognitive performance and cellular events in elderly brains. These data suggest that APAP-containing pharmacological interventions may foreshadow the elevated risk of neuropsychiatric disorders that afflict elderly populations.

## Introduction

Acetaminophen (APAP) is the leading cause of acute liver failure (ALF) in North America and Europe^1–3^. In the United States alone, this form of hepatotoxicity accounts for approximately 20% of liver transplant cases and over 600 deaths a year^1,4,5^. This is due in part to the notorious omnipresence of APAP in pharmaceutical formulations, as evidenced by more than 500 over-the-counter and prescription medicines^6^. The metabolite is known to readily cross the blood–brain barrier (BBB)^7,8^ and promote analgesic and antipyretic effects through mechanisms not yet fully elucidated, but which appear to involve complex metabolic pathways (e.g., prostaglandin synthesis and nociceptive pathways)^9^. Excess APAP exposure, resulting from either the frequent use of recommended concentrations (≤ 4000 mg per day in adults; per the US Food and Drug Administration) or the single intake of a very high dose, causes major deleterious effects on the liver that have been extensively characterized over several decades^10–12^. Strikingly, this form of hepatotoxicity is also associated with cerebral abnormalities such as anxiety, disorientation, sluggish speech, and even loss of consciousness and coma that typically manifest in the form of hepatic encephalopathy^13–17^. The link between APAP and neurological impairments remains poorly understood, bolstering the growing concern over the metabolite’s enormous pharmaceutical prevalence and ease of access to populations that include the elderly^6^. It is important to highlight that elderly persons are at notable risk of the medication overload that elevates the likelihood of APAP exposure^18^, the compromised liver function that facilitates accumulation of toxic metabolites^19^, and the aging-associated disorders, such as Alzheimer’s disease (AD), that elevate the brain’s susceptibility to and severity of dyshomeostasis events^20^.

APAP metabolism is chiefly performed by the parenchymal liver cells, hepatocytes, and results in the formation of various byproducts including *N*-acetyl-*p*-benzoquinone imine (NAPQI), which is highly cytotoxic^21,22^. The antioxidant molecule glutathione (GSH) is tasked with the neutralization of this toxic byproduct to mitigate such deleterious effects. However, excess APAP intake results in the rapid depletion of cellular GSH stores, followed by hepatic damage as hepatocytes undergo cell death^23–25^. In contrast to knowledge of APAP’s effects in the periphery, little is known about its contributions in the brain because experimental studies on the topic remain relatively scarce. Still, these efforts show that APAP intoxication is associated with cerebral abnormalities that vary depending on factors beyond the metabolite’s concentration, including the subject’s age^26–32^. To our knowledge, experimental studies so far have examined rodent brains only up to adult life stages (typically, ≤ 6 months of age). And thus, there is desperate need to elucidate the neurological effects of APAP in elderly hosts, who are likely to be exposed since an earlier age^6,33,34^ and correspond to the subgroup at highest risk of various relevant factors as noted earlier (APAP overexposure^18^, NAPQI accumulation^19^, and vulnerable to brain abnormalities^20^).

In this study, we submitted mice to monthly episodes of APAP intoxication starting as a young adult (90-days-old) until an elderly age of 420 days. Following this, we performed cognitive analyses and examined pathology in the brain and periphery of elderly mice. Here, we report that at a late life stage, history of APAP intoxication is associated with accelerated worsening of non-spatial memory deficits, but not spatial memory performance. Further, one month after the final episode of APAP intoxication, elderly mice brains showed elevated glial burden without the increase of inflammatory cytokines and chemokines, blood–brain barrier (BBB) disruption, and cholesterol accumulation, among other biomarkers. Ultimately, this experimental study crucially advances knowledge of the long-term neurological effects of recurrent APAP exposure in the elderly.

## Methods

### Animals and treatments

129S6/SvEvTac mice (Taconic Biosciences, Rensselear, NY, USA) were housed at facilities with controlled room temperature (25 °C), humidity (0–60%), and 12-h light/dark cycles. A standard food diet and water were provided ad libitum. Mice were randomly assigned to PBS (*n* = 7 males; *n* = 8 females) and APAP (*n* = 6 males; *n* = 6 females) groups, and each cage consisted of up to 5 animals in the same group. Male and female mice underwent monthly intraperitoneal (i.p.) injection of either saline vehicle (PBS) or APAP (300 mg/kK body weight, Sigma-Aldrich (St. Louis, MO, USA; CAS No: 103-90-2)) starting at 90 days of age. Prior to each injection, animals were fasted for 15 h with free access to water. Treatments were performed by a single researcher and continued until mice were 420-days-old. One month later, the brains and livers from mice were collected. Blood samples from animals were acquired via retroorbital procedure at 48 h following each injection, as well as at the experimental endpoint (450 days of age). Experiments were performed in accordance with the Guidelines by the NIH Office of Laboratory Animal Welfare regarding the care and use of animals for experimental procedures, and following the recommendations stated in the ARRIVE guidelines (https://arriveguidelines.org/). The experiments were approved by the UTHealth-Houston Animal Welfare Committee (AWC) under animal protocol number 21-0065.

### Serum isolation and ALT activity quantification

Collected blood samples were placed in 1.5 mL centrifuge tubes that were subsequently submitted to overnight incubation at 4 °C and allowed to clot. Following incubation, centrifuge tubes containing samples underwent centrifugation at 10,000×*g* for 10 min and the supernatant (serum) removed. Sera were stored at -80 °C until biochemical procedures, apart from ALT activity analysis, were performed. ALT measurements were conducted in fresh specimens using an ALT Activity Assay (Sigma-Aldrich, St. Louis, MO, USA; SKU: MAK052-1KT). The ALT Activity Assay employs a coupled enzyme reaction that results in a colorimetric/fluorometric product proportional to the amount of pyruvate that is generated. The ALT activity assay was performed according to the manufacturer’s protocols for colorimetric detection with some alterations. In summary, using a 96-well microplate, 20 μL of serum samples were added to separate wells containing the Master Reaction Mix. Next, the microplate was placed in a SpectraMax iD3 Multi-Mode Microplate Reader (Molecular Devices, San Jose, CA, USA) at 37 °C, followed by horizontal shaking for 30 s and subsequent reading of absorbance at 570 nm every minute until the value of the most active sample surpassed that of the highest standard. Standard curve analysis of the absorbance values was used to calculate the total amount of pyruvate in each well as a proxy to ALT activity levels.

### Novel object recognition and object location tests

The open-field chamber (60 × 40 × 50 cm) served as the arena for both novel object recognition (NOR) and object location test (OLT) analyses at 436 and 443 days of age, respectively. For both NOR and OLT assays, habituation and training sessions occurred on day 1, and was followed by a test trial at day 2. On day 1, mice underwent a single habituation session wherein animals were allowed to freely explore the arena for 5 min (both NOR and OLT). One hour later, two identical objects were added to the arena. To avoid multiple exposures to a single familiarization environment, these objects’ shape and positioning on the training arena varied depending on the memory test (NOR: round shape, positioned at quadrants 1 and 3; OLT: rectangle shape, positioned at quadrants 2 and 4). The animals were given 5 min to explore the training arena. On day 2 (test trial), NOR involved providing the mice 5 min to explore the same arena while either replacing one of the previous two items with a novel object of similar size but different shape (square), whereas OLT included relocating one of the previous two items to a different quadrant in the arena. The objects and arena were cleaned between trials to avoid the accumulation of odors. A single analyst performed all memory tests to avoid potential differences in terms of animals’ anxiety. Each mouse was evaluated for visible signs of anxiety and related behavioral changes that might confound results in the testing phase during the habituation and training stages. Animals in the control and test groups showed no visible (qualitative) differences in exploratory drive, lethargy, and thigmotaxis during all phases of memory tests. In addition, mice did not exhibit signs of neophobia while in the training for NOR and OLT procedures. The time spent interacting with either the novel or relocated object was recorded using TopScan analysis software (CleverSys, Inc., Reston, VA, USA), and the associated object discrimination index was calculated via the following formula: B ÷ (A + B); (A = time spent exploring the object that is unchanged; B = time spent exploring the novel/relocated object).

### Tissue collection and total protein concentration estimation

Mouse brains were collected and the right and left hemispheres isolated. For the interrogation of cerebral pathology via immunohistochemistry (IHC), the left hemispheres were fixed in 10% neutral buffered formalin (Thermo Fisher Scientific, Waltham, MA, USA; Cat No: 22-220682) for 48 h. Next, left hemispheres were washed for 10 min three times in PBS at room temperature, followed by cryopreservation via placement in a 15 mL conical tube containing 30% sucrose in PBS until the tissues sunk to the bottom. Next, samples were snap-frozen using dry ice powder and stored at − 80 °C until sectioning for immunofluorescence (IF) analysis. To examine tissue pathology using biochemical procedures, the right hemisphere of the brains and right liver lobes were collected. The isocortex and hippocampus (10% w/v) regions of the right brain hemispheres were dissected, and alongside the liver lobes, submitted to homogenization in PBS solution containing a protease inhibitor cocktail (Sigma-Aldrich, St. Louis, MO, USA; SKU: 11697498001) in a Precellys Evolution Touch homogenizer (Bertin Technologies, Rockville, MD, USA; Cat No: P002511-PEVT0-A.0). All homogenates were frozen in liquid nitrogen and stored at − 80 °C until biochemical analyses. A BCA^®^ protein assay kit (Thermo Fischer Scientific, Waltham, MA, USA; Cat No: 23225) was used to quantify total protein content for the normalization of results where applicable.

### Immunohistochemical evaluation of neuropathology

The brain sections were cut to 40 µm via frozen sectioning using a Leica SM2010R sliding microtome (Leica Biosystems, Deer Park, IL, USA). Sections were blocked with 5% goat serum in PBS containing 0.2% v/v Triton X, followed by overnight incubation with unconjugated, rabbit polyclonal antibodies against the ionized calcium-binding adapter molecule 1 (IBA-1; FUJIFILM Wako, Prod No: 019-19741) and the glial fibrillary acidic protein (GFAP; Abcam, Cat No: ab7260) at a dilution of 1:1000, or with an identical concentration of an unconjugated, rabbit monoclonal antibody against transmembrane protein 119 (TMEM119; Abcam, Cat No: ab209064). After incubation, sections were washed with PBS and then incubated with Alexa Fluor Plus 594 (1:500, Invitrogen, Cat. No: A11012) for immunofluorescence (IF) analysis. Microscopy was performed using a Leica DMi8 microscope (Leica Biosystems, Waltham, MA, USA), and staining in the mouse isocortex and hippocampus regions were quantified using the ImageJ software (https://imagej.nih.gov/ij/).

### Multiplex assay to quantify cytokines and chemokines

The xMAP technology (Luminex Corp.) employs a sandwich immunoassay with fluorescent bead-based technology to interrogate the concentrations of up to 500 target analytes in a single sample. A commercially available murine Bio-Plex Pro assay kit (Bio-Rad Laboratories, Inc., Hercules, CA, USA; #M60009RDPD) was acquired to quantify the brain and serum levels of up to 23 cytokines and chemokines. These inflammatory molecules included eotaxin, granulocyte colony-stimulating factor (G-CSF), granulocyte–macrophage colony-stimulating factor (GM-CSF), interferon (IFN)-γ, keratinocyte chemoattractant (KC), monocyte chemoattractant protein (MCP)-1, macrophage inflammatory protein (MIP)-1α, MIP-1β, regulated on activation, normal T cell expressed and secreted (RANTES), tumor necrosis factor (TNF)-α, and interleukin (IL)-1α, -1β, -2, -3, -4, -5, -6, -9, -10, -12(p40), -12(p70), -13, and -17A. Multiplex analysis was performed according to the manufacturer protocols. In summary, samples underwent incubation with the magnetic antibody-coated beads for the capture of target analytes. Next, samples were exposed to biotinylated detection antibody and streptavidin–phycoerythrin (PE) fluorescent reporter to permit quantification of target cytokines and chemokines using a Bio-Plex suspension array system (Bio-Rad Laboratories, Hercules, CA, USA).

### Liver and serum low density lipoprotein receptor-related protein 1 (LRP-1) quantification

Measurement of LRP-1 levels in tissue homogenates and sera was performed using a Mouse Low Density Lipoprotein Receptor Related Protein 1 ELISA Kit (MyBioSource, San Diego, CA, USA; Cat No: MBS450380), as recommended by the manufacturer. In summary, 100 μL of samples were added into separate wells of the ELISA plate, covered with a plate sealer, and allowed to incubate for 2 h at 37 °C. After, contents in each well were removed and replaced with 100 μL of Detection Reagent A prior to incubation for 1 h at 37 °C while covered with plate sealer. Next, the wells were washed with wash buffer, followed by addition of 100 μL of Detection Reagent B working solution to each well and subsequent incubation for 1 h at 37 °C after covering with plate sealer. Next, the ELISA plate was washed with wash buffer and 90 μL of Substrate Solution was added to each well, covered with plate sealer, and allowed to incubate for 20 min at 37 °C while in the dark. Finally, 50 μL of Stop Solution was added to each well and mixed, followed by measurement of absorbance at 450 nm in a SpectraMax iD3 Multi-Mode Microplate Reader (Molecular Devices, San Jose, CA, USA). Results were calculated by standard curve analysis.

### Free cholesterol analysis

We used the Amplex^®^ Red Cholesterol Assay kit (Thermo Fisher Scientific, Waltham, MA, USA; Cat No: A12216), a fluorometric procedure based on an enzyme coupled reaction to quantify levels of free cholesterol in tissues. Assays were performed according to manufacturer’s protocols. Briefly, 50 μL of samples were placed in separate wells of a 96-well microplate containing an equal volume of the Amplex^®^ Red working solution without cholesterol esterase to measure free cholesterol levels. The reactions were allowed to incubate in the dark at 37 °C for 30 min, during which fluorescence was measured in a SpectraMax iD3 Multi-Mode Microplate Reader (Molecular Devices, San Jose, CA, USA) using excitation in the range of 530–560 nm and emission detection at 590 nm. Fluorescence values at various time points were used to calculate levels of free cholesterol in samples via standard curve analysis.

### Statistical analyses

Results are presented as mean ± SD. Data were analyzed using Student’s *t*-test to compare two groups, as well as via two-way repeated-measures ANOVA or mixed effects model followed by Tukey’s test for post hoc comparisons where appropriate. Statistical significance was set as *p* < 0.05. All statistical calculations were done using GraphPad Prism 8.0 (GraphPad Software Inc., La Jolla, CA, USA).

## Results

### Modeling the long-term effects of recurrent APAP intoxication until an elderly age

**Figure 1:**
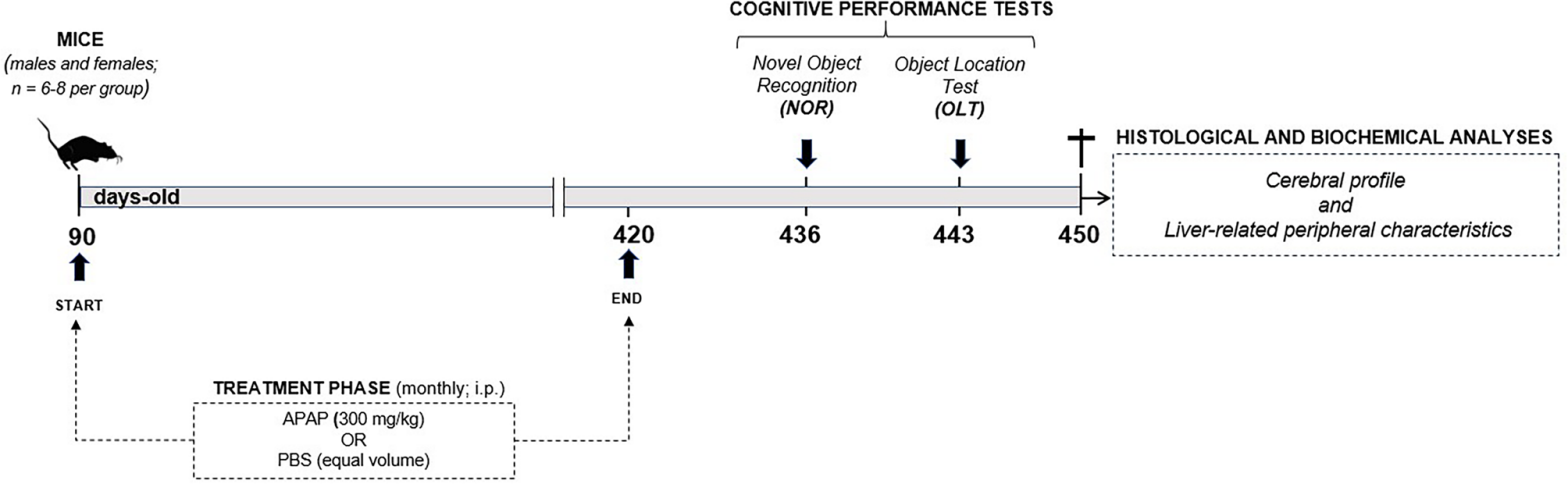
Schematic depiction of research methodology. Representative image outlining the experimental study design.

APAP intoxication can occur intentionally (suicide effort) or unintentionally (therapeutic misuse and/or unsupervised prolonged/frequent administration)^3,35,36^, which is notable because the latter type is also a key driver of the medication overload that plagues elderly populations^18,37^. In this sense, such individuals are at relevant risk of exposure to the metabolite and yet, to our knowledge, have not been evaluated in the context of associated cerebral abnormalities under experimental conditions. Because frequent APAP exposure can start at any age^3,14,38,39^, we designed an experimental study to interrogate the neurological effects of the metabolite in elderly animals with history of excess intake (Fig. 1). Briefly, adult mice, male and female, underwent repeated exposure to 300 mg/Kg of APAP, a dose recommended for liver injury in both sexes as evidenced by elevated serum levels of ALT activity^40^—and confirmed by us in pilot trials (data not shown). Moreover, this metabolite concentration is equivalent to an approximately 3375 mg daily dosage for a 70 Kg human subject that is within recommended guidelines for human adults (≤ 4000 mg/kg every 24 h; per the US Food and Drug Administration); and thus, more closely reflects unintentional rather than intentional hepatic injury, which is important since the former is linked to greater morbidity and mortality than the latter^35,36^. To mimic such history of liver damage until an elderly life stage, treatments were performed monthly via i.p. injection to ensure consistent intake of a 300 mg/Kg dose of APAP, or saline control, starting at 90 days of age and lasting a period of nearly one year (330 days), corresponding to an age of 420 days. To confirm the hepatic injury status at designated time points, we assayed serum levels of ALT activity through a biochemical procedure (see Supplementary Fig. S1). The animals underwent non-spatial and spatial memory analyses at 436 and 443 days of age, respectively, whereas cerebral and peripheral pathologies were evaluated via IHC and biochemical analyses at one month after the final treatment, when mice were 450-days-old.

Ultimately, we observed high mortality among APAP-treated male subjects (see Supplementary Fig. S1; 50%, *n* = 3 of 6); and in turn, by endpoint, conclusive evaluation of this group was not feasible since few tissues were available for analyses. Thus, hereafter we communicate the cerebral and peripheral findings in elderly females only, as associated findings have both high validity and novelty.

### Elderly female mice exhibit reduced hepatic levels of LRP-1 long after recurrent APAP intoxication

**Figure 2:**
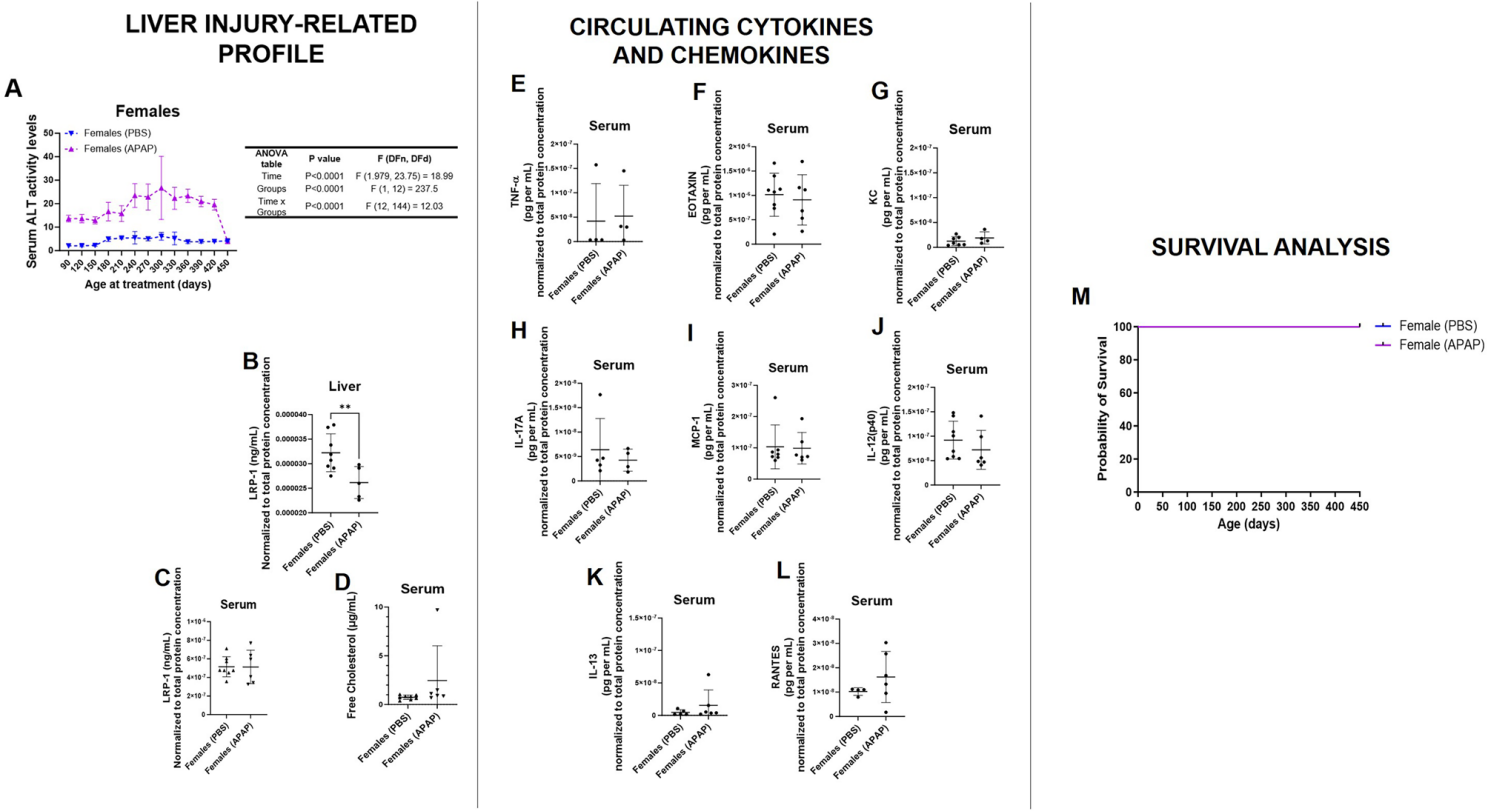
Evaluation of liver and inflammatory markers in APAP and PBS treated mice in blood and liver. At endpoint, elderly female mice with history of APAP intoxication exhibited reduced levels of hepatic LRP-1, but no differences in cholesterol accumulation, peripheral inflammation, and mortality compared with PBS-treated counterparts. Female mice sera collected each month since start of treatment was evaluated for ALT activity to query circulating levels of the liver injury marker, and a graph of results is shown alongside a table outlining test details of 2-way repeated measures ANOVA (extended details are seen as Supplementary Table S1) (**A**). Quantification of LRP-1 levels in liver homogenates (**B**) and sera (**C**) from mice. Circulating concentrations of free cholesterol (**D**), and inflammation modulators (**E**–**L**). Statistical analyses include 2-way repeated measures ANOVA (**A**) and one-tailed unpaired Student’s *t*-test (**B**–**L**). *p < 0.05. *LRP-1* liver-related receptor 1, *KC* keratinocyte chemoattractant, *MCP-1* monocyte chemoattractant protein-1, *RANTES* regulated on activation, normal T cell expressed and secreted, *TNF-α* tumor necrosis factor-α, *(IL)-12(p40), -13, and -17A* interleukin.

We used an ALT activity kit to measure the circulating levels of this established biomarker of hepatic injury^41^. Liver status was queried at each month starting at onset of treatment until the experimental endpoint. A two-way repeated measures ANOVA revealed a statistically significant interaction between treatment groups and time on the serum levels of ALT activity (Fig. 2A, see Supplementary Table S1; F(12, 144) = 18.99, *p* < 0.0001). Specifically, we found that hepatic damage was higher in females treated with APAP compared to the PBS group throughout the course of treatment (monthly; 90–420 days of age), but not at the experimental endpoint (450 days of age) to suggest that associated toxicity occurred in acute manner. This is further supported in the fact that monthly ALT measurements were taken 48 h after APAP administration, while the final measurement occurred 28 days after the last treatment. Of note, an identical pattern of longitudinal liver response to treatments was observed even when associated data from male groups were included (see Supplementary Fig. S1 and Supplementary Table S2).

Reports indicate that liver injury influences the major hepatic function modulator LRP-1^42,43^, followed by the disruption of downstream processes such as cholesterol homeostasis and modulation of peripheral inflammation^44^. In turn, we next used biochemical procedures to examine these features at one month since the final treatment of mice. We observed significantly reduced levels of LRP-1 in liver homogenates from the test group, relative to controls (Fig. 2B; *p* = 0.0070); however, no differences were observed in the soluble form of this receptor (sLRP-1) (Fig. 2C). Moreover, mice from both groups exhibited non-significantly different levels of serum free cholesterol (Fig. 2D) as well as various cytokines and chemokines (Fig. 2E–L). These findings reveal that at one month since the last episode of recurrent APAP intoxication, elderly female mice do not exhibit either free cholesterol accumulation, a known consequence of frequent liver damage^45^, or peripheral inflammation. Finally, despite the extensive duration of treatment and the subjects’ considerably older age by endpoint, no mortality was observed among control and test female groups (see Fig. 2M and Supplementary Fig. S1).

### Recurrent exposure to APAP exacerbates non-spatial memory deficits in elderly female mice, but not the spatial memory performance

**Figure 3:**
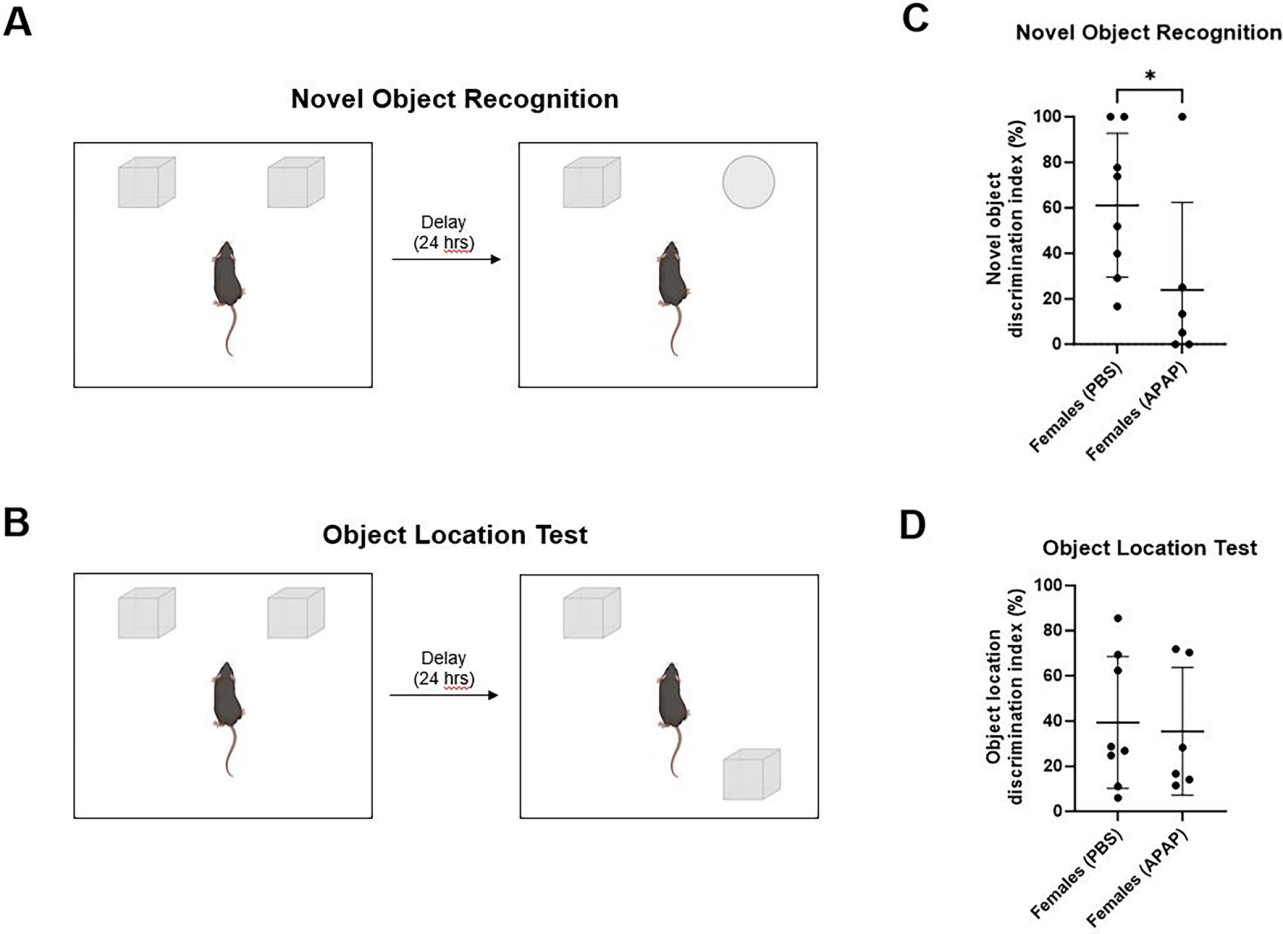
APAP administration induces non-spatial memory decline. In elderly female mice, recurrent exposure to APAP starting at an earlier life stage accelerates non-spatial memory deficits, but not spatial memory performance. Female mice underwent monthly treatment with saline vehicle (PBS) or APAP starting at 90 days of age until 420-days-old (*n* = 8 and 6, respectively). At 436 days of age, animals were subjected to the novel object recognition assay (**A**), and 1 week later, the object location test (**B**). Indices showing the results of NOR test (**C**) and OLT assay (**D**) evaluation of PBS- and APAP-treated mice. One-tailed unpaired Student’s *t*-test. *p < 0.05. Images created with Biorender.com.

Prior studies show that excess APAP intake can result in diverse types of cognitive impairment in mice^27,30^. To determine the effect of recurrent APAP intoxication on non-spatial and spatial memory performance, elderly mice were submitted to NOR and OLT analyses, respectively, as depicted in the Fig. 3A,B. In the former test, APAP was associated with significantly lower novel object discrimination index scores compared to controls (Fig. 3C; *p* = 0.0349), which indicated that age-related nonspatial memory decline had been aggravated. In contrast, OLT analysis showed that the object location discrimination index scores were comparable between control and test groups (Fig. 3D); and thus, revealed that in elderly female mice, a history of APAP intoxication is not also linked to worsened spatial memory deficits.

All mice included in this study were assessed for visible (qualitative) differences in exploratory drive, lethargy, neophobia and thigmotaxis during all phases of memory tests while in the working arena. Although no obvious differences in these behaviors were observed between the groups, we cannot exclude that specifically designed tests would unveil other such behavioral abnormalities caused by chronic APAP treatments.

### Long-term effects of recurrent APAP intoxication on elderly female mice brains include elevated levels of astrocyte and microglia, but not other typical drivers of cognitive impairment

**Figure 4:**
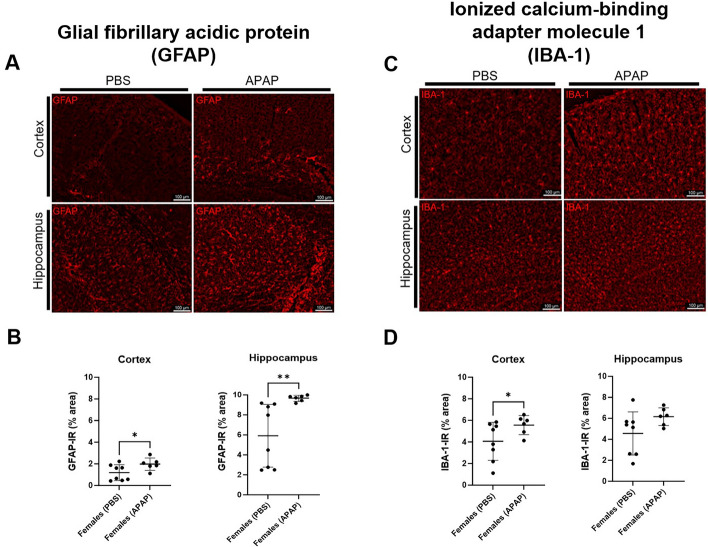
Repetitive APAP treatment induces long-term increase in brain gliosis. Elderly female mice displayed elevated glial burden at one month following the last episode of APAP-induced liver injury. Female mice were subjected to monthly intraperitoneal injection with saline vehicle (PBS) or APAP starting at 90 days of age until 420-days-old (*n* = 8 and 6, respectively). At 450 days of age, mice brains were isolated and submitted to immunofluorescence (IF) analysis. Representative images of IF staining of frozen brain sections from PBS- and APAP-treated female mice for the astrocyte marker, GFAP (**A**), and the microglia/macrophage marker, IBA-1 (**C**). Scale bars represent 100 µm. ImageJ analysis was used to quantify cortical and hippocampal levels of astrocytes (**B**) and microglia (**D**) in mice brains. One-tailed unpaired Student’s *t*-test. *p < 0.05, **p < 0.01.

**Figure 5:**
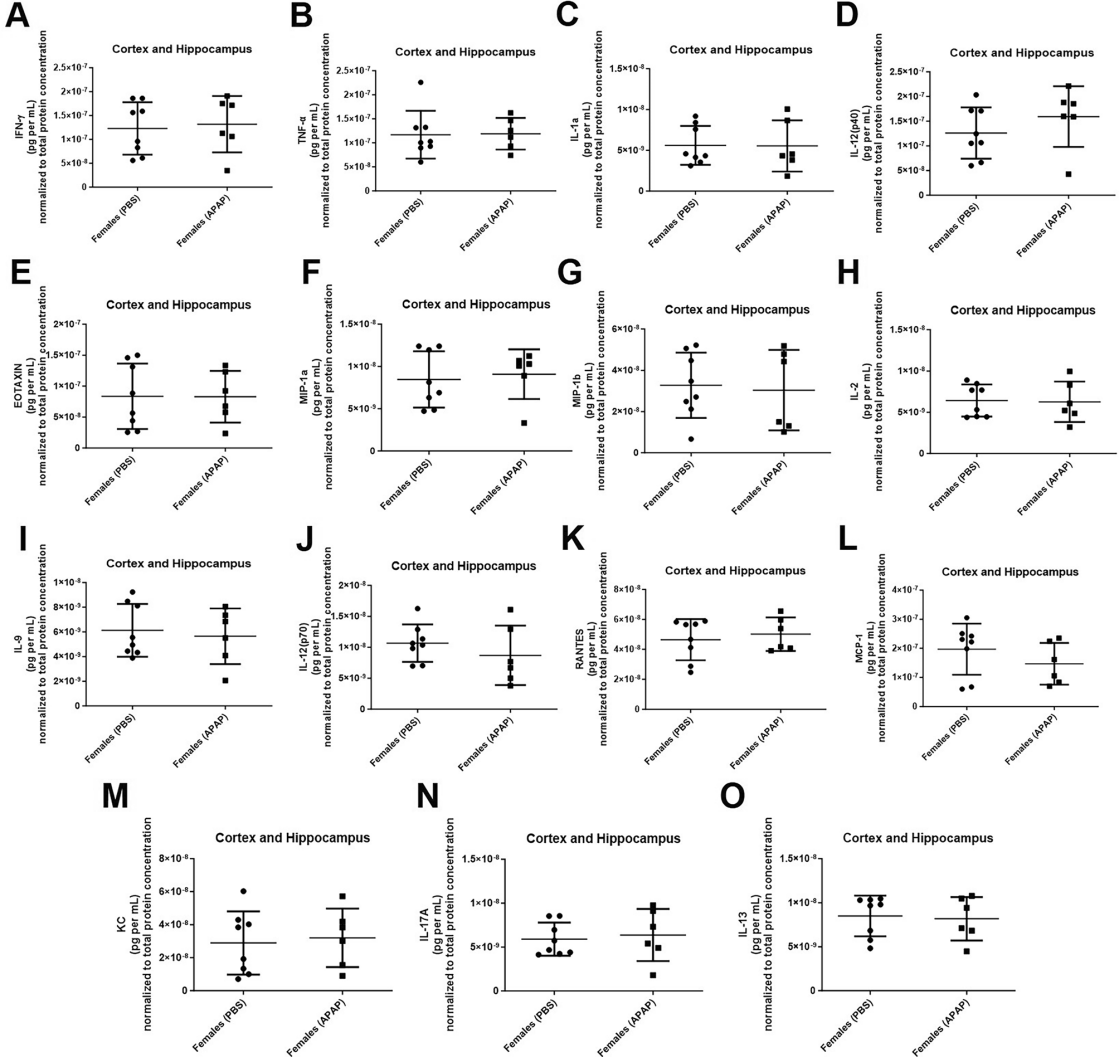
Cytokines and chemokines levels in the brains of APAP treated mice. At endpoint, brains from elderly female mice with and without history of recurrent APAP exposure exhibited comparable levels of numerous cytokines and chemokines. Female mice underwent monthly treatment with saline vehicle (PBS) or APAP starting at 90 days of age until 420-days-old (*n* = 8 and 6, respectively). Multiplex analysis of sera from mice at 450 days of age to quantify levels of various cytokines and chemokines (**A**–**O**). One-tailed unpaired Student’s *t*-test. *p < 0.05. *IFN-γ *interferon-γ, *KC* keratinocyte chemoattractant, *MCP-1* monocyte chemoattractant protein-1, *MIP-1α*, *MIP-1β* macrophage inflammatory protein-1α, -1β, *RANTES* regulated on activation, normal T cell expressed and secreted, *TNF*-*α* tumor necrosis factor*-*α, *IL-1α, -1β, -2, -9, -10, -12(p40), -12(p70), -13, and -17A* interleukin.

Elderly hosts are susceptible to experience cognitive decline concomitant with a shift to a pro-inflammatory state in the brain, relative to younger counterparts. Typically, this occurs via the modulation of astroglia and/or microglia burden to upregulate the expression of various cytokines and chemokines^46^. To evaluate the long-term effect of recurrent APAP exposure on these phenotypes at late age, we used IF staining to measure astrocyte and microglia/macrophages concentrations, as well as performed biochemical quantification of numerous cytokines and chemokines using homogenate comprised of the isocortex and hippocampus (the brain regions most relevant to spatial and non-spatial memory performance^47,48^). On the one hand, we found that history of APAP intoxication was associated with significantly increased levels of the astroglial marker GFAP (glial fibrillar acidic protein) in the isocortex and hippocampus, relative to controls, (Fig. 4A,B; *p* = 0.0239 and 0.0067, respectively). On the other hand, the marker for microglia/macrophages IBA1 was significantly elevated in the isocortex (Fig. 4C,D; *p* = 0.043). As IBA1 is not a microglia-exclusive marker, we measured the staining of an additional and more specific microglial marker (the transmembrane protein 119, TMEM119) in the brains of both control and experimental groups. Notably, the results of this analysis differed from those obtained measuring IBA1, as APAP treatments were associated with elevated levels of TMEM119 in the hippocampus instead of the isocortex (Fig. S2 and Fig. 4). Nevertheless, both IBA1 and TMEM119 results are consistent with APAP inducing neuroinflammatory phenotypes. Yet, biochemical analysis indicated that at endpoint, the cerebral concentrations of cytokines and chemokines including IFN-γ and TNF-α, among many others, were comparable between PBS and APAP groups (Fig. 5).

**Figure 6:**
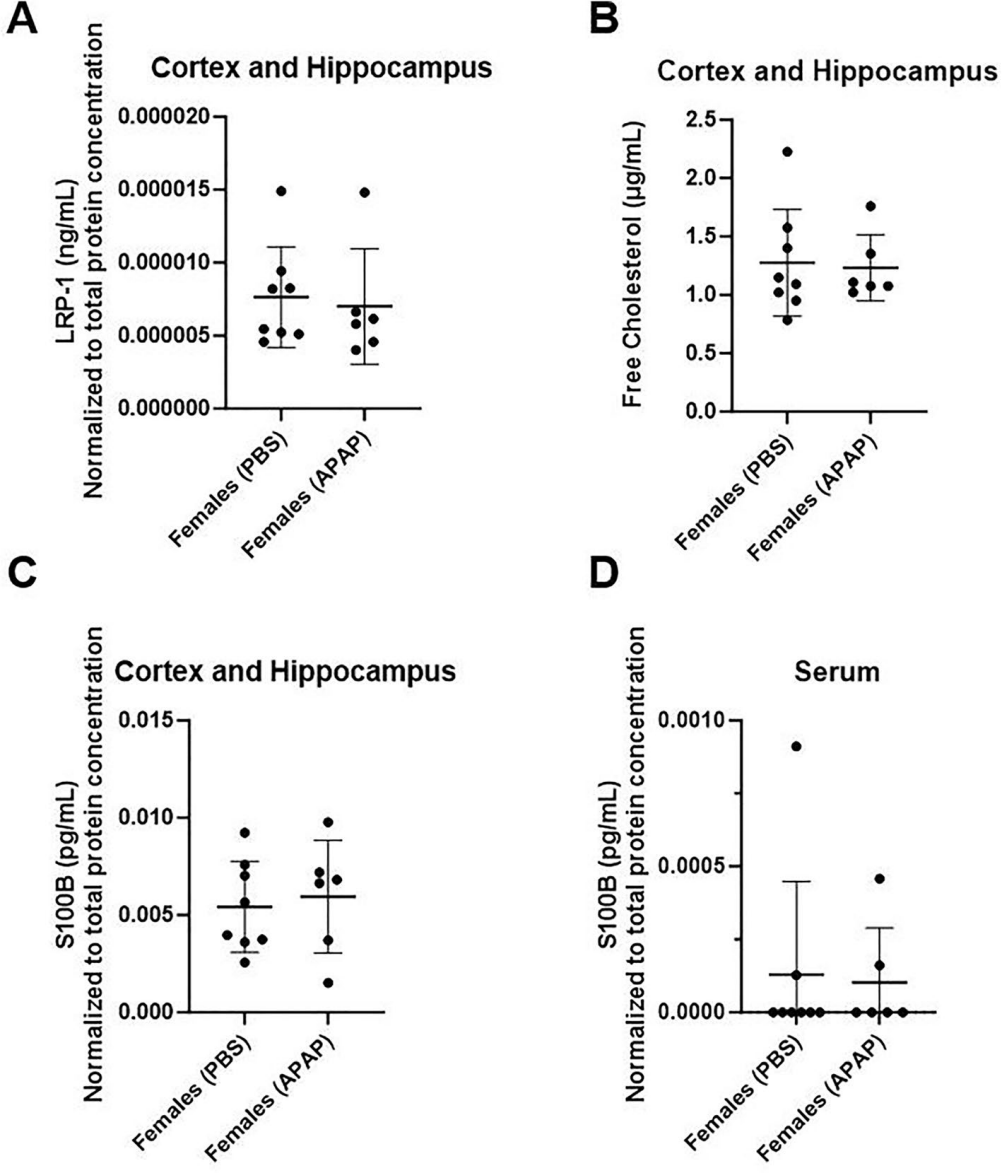
LRP-1, free-cholesterol and s100b levels in the brains of APAP- and PBS- treated mice. At endpoint, elderly female mice exhibit comparable cerebral levels of LRP-1, and free cholesterol, as well as identical neuronal and blood–brain barrier integrity. Female mice underwent monthly treatment with saline vehicle (PBS) or APAP starting at 90 days of age until 420-days-old (*n* = 8 and 6, respectively). Brain homogenates from mice at 450 days of age were collected and submitted to biochemical quantification of LRP-1 (**A**), free cholesterol (**B**), and s100b levels (**C**). The latter analyte was also queried in sera from these animals (**D**). One-tailed unpaired Student’s *t*-test. *p < 0.05.

Hepatic abnormalities are demonstrated to promote the cerebral accumulation of cholesterol and subsequently aggravate cognitive status and related neuropathology^49,50^. As in the liver, LRP-1 crucially modulates the homeostasis of lipids in the brain including cholesterol, which is crucial to myelin development and influences processes such as memory performance and glial status^51,52^. Considering our observation that APAP intoxication is linked with reduced levels of hepatic LRP-1 (Fig. 2B), we next assayed the cerebral concentration of this receptor as well as that of free cholesterol. Biochemical evaluation indicated that at endpoint, all elderly female subjects showed similar levels of LRP-1 and free cholesterol in the brain (Fig. 6A,B) to suggest that cholesterol dyshomeostasis is not a long-term effect of recurrent APAP intoxication in this host. Further, we assayed s100b concentrations in the brain and serum to query neuronal and BBB integrity, respectively, as this protein also links liver damage-driven cognitive impairment and increased glial burden^53,54^. We found that APAP and PBS groups displayed comparable cerebral and circulating levels of s100b (Fig. 6C,D) to indicate no differences in these phenotypes at endpoint.

Together, these data highlight that in elderly female mice, the long-term effect of recurrent APAP intoxication since an early age includes the elevated burden of astrocytes and microglia, but not other cerebral abnormalities that typically link peripheral dyshomeostasis events with cognitive deficits.

## Discussion

In the U.S. and many other Western nations, APAP intoxication is the primary cause of ALF^1,2,5^. Strikingly, this condition can result in hepatic encephalopathy, a disorder characterized by an array of debilitating cognitive deficits and recurrence^4,13–15^. The true incidence and prevalence of this disease is not yet determined; however, owing to APAP’s status as the most common drug ingredient in America^6^, the gap in knowledge of associated cellular and molecular events in the brain represents a relevant risk to populations. Notably, this concern is bolstered by other relevant factors. First, frequent APAP intake can promote age-related deleterious effects in the brain and periphery^11,27,55^. Second, recurrent APAP exposure can occur starting at any age^3,14,38,39^, which is particularly concerning for elderly hosts due to compounding risk factors that include the increased susceptibility to overmedication^18^ and the age-related impairment in hepatic and cerebral functions^19,20^. Thus, there is need for experimental efforts that query the neurological effects of recurrent APAP intoxication while accounting for yet evaluated, but translationally relevant parameters such as elderly subjects. In this study, male and female mice at 90 days of age were submitted to monthly i.p. injections of a single dose of APAP at 300 mg/Kg, or PBS. Treatments were performed until animals reached an age of 420 days, and weeks after, we interrogated cognitive performance as well as cellular and molecular events in the brain and periphery to determine associated long-term effects. To our knowledge, this work first reports the neurological effects of APAP in elderly subjects that were repetitively treated under experimental conditions.

We found that the test male group, but not females, exhibited high mortality. This observation is consistent with the well-established sexual dimorphism in rodent liver tolerance to APAP (females > males)^56,57^. The accepted mechanism of action underlying the sex-specific rodent liver response to APAP is the faster rate of de novo GSH synthesis in females compared to males. The GSH conjugate formation with APAP mitigates the saturation and exhaustion of sulfation and glucuronidation pathways following accumulation of the metabolite, which would otherwise lead to mitochondrial disruption and cellular death^10,58^. This key role of GSH is best evident in clinical settings via the successful use of *N*-acetyl-l-cysteine as the standard of care for APAP overdose^59,60^, notwithstanding an array of rodent studies^61–63^. In our work, the elevated fatality in male groups was highly consequential due to the increased risk of mortality-driven bias in the data—that arises when comparing a group comprised of the individuals most tolerant to treatment (APAP-treated males) with another that consists of the individuals both least and most tolerant to treatment (APAP-treated females). Because of this major confounding variable, and the insufficient number of tissues from APAP-treated males available for analyses to allow conclusive evaluation, only female subjects underwent the cognitive tests and the characterization of cerebral and peripheral pathology at endpoint.

Our data show that liver damage occurred at designated injection time points but, despite the extensive history of APAP intoxication, at 1 month since the final episode control and test female groups exhibited comparable levels of serum ALT activity. This observation suggests that associated liver injury had been acute, which is consistent with the literature on APAP^3,12^, but is nonetheless surprising due to the considerable duration of treatment. Interestingly, at endpoint, we found that APAP-treated female mice displayed lower levels of LRP-1 in the liver to implicate chronic deficits on downstream processes including cholesterol homeostasis and peripheral inflammation^44^. And yet, subsequent analyses of associated biomarkers revealed no such differences between control and test females. Indeed, we observed similar circulating concentrations of sLRP-1, various pro-inflammatory cytokines including TNF-α, and free cholesterol, which suggested that disruption of circulating ligand trafficking^64^, peripheral inflammation^65^, and cholesterol homeostasis^66^, respectively, did not occur at endpoint despite the marked effects on hepatic LRP-1 concentrations. Perhaps, these findings are explained by the well-established acute nature of APAP-induced hepatotoxicity and elevated tolerance of female mice. Such a premise agrees with our earlier observation that hepatotoxicity occurs at all designated time points for injection of the metabolite but not at endpoint, which corresponds to 28 days since the final treatment. Still, it is interesting that repeated APAP administration nonetheless correlated with worsened non-spatial memory performance to implicate the accelerated disruption of lateral entorhinal cortical inputs to the hippocampus^67^. Moreover, test mice did not exhibit differences in spatial memory deficits, which are more largely modulated by the medial entorhinal cortical inputs to the same brain region^68^. Therefore, our data reveal that at an elderly life stage, recurrent APAP intoxication exacerbates non-spatial memory, but not the spatial memory performance of female mice. Future experiments in more sensitive male mice and human subjects will clarify whether long-term APAP treatments are linked to effects in these individuals.

Because age-related cognitive performance is linked to the status of glial cells^20^, we next queried associated biomarkers in elderly females and found that brains derived from the APAP group displayed increased levels of astrocyte markers in the isocortex and hippocampus, as well as elevated hippocampal presence of microglia. These data are in line with the earlier finding of non-spatial memory impairment. Although this increase in glial burden persisted long after the final episode of recurrent APAP intoxication, female mice were not associated with altered levels of immune modulators such as TNF-α and IFN-γ, among many others. Thus, indicating that inflammation is not robust in brains from elderly female mice with history of liver injury. We did not query cytokine concentrations at earlier time points, and therefore cannot rule out the possibility that robust neuroinflammation occurred throughout the course of treatment or acutely after APAP administration to explain the cognitive deficit we observed. Similar consideration is given to other relevant cerebral biomarkers measured only at endpoint. For example, BBB disruption, yet another feature known to link hepatotoxicity to changes in glial burden and cognitive impairment^49,54^, was also not evident at one-month post hepatotoxicity. Further, although reports show that cerebral levels of LRP-1 negatively correlate with the severity of brain dyshomeostasis^51,69^, our work reveals that in elderly female mice with a history of APAP intoxication, the cerebral concentrations of this receptor are comparable between the control and test groups. This observation contrasts with our findings in the liver, where LRP-1 was decreased. Still, we found no changes to brain levels of free cholesterol, which is notoriously cytotoxic^45^ and links LRP-1 to cerebral abnormalities^49–52^. This result is consistent with the evaluation of LRP-1 in the brain. Finally, all animals showed identical levels of neuronal and BBB integrity to corroborate the striking absence of other putative drivers of the non-spatial memory deficit and elevated glial burden we observed.

Collectively, this study complements previous experimental efforts on the topic, as the overwhelming majority of such reports do not congruently examine pathology in the periphery, nor include female subjects, which crucially limits knowledge of how periphery- and sex-based factors contribute to the brain’s response to APAP. In the few studies that interrogate cerebral effects, APAP intoxication is associated with spatial and non-spatial memory deficits alongside elevated levels of oxidative stress and gliosis^26–32^; and intriguingly, sex-specific effects are not observed^32^. Such observations implicate some differences between APAP accumulation-induced pathways and/or molecules in the brain compared to the liver. Considering that even the beneficial neurological effects of APAP remain poorly elucidated, further investigations on the topic are desperately needed to improve mechanistic understanding.

Reports in humans and rodents indicate that elderly subjects are associated with higher serum levels of APAP, relative to those at younger ages^70,71^. This is significant because, as further detailed below, the peripheral administration of APAP is linked to concentration-dependent in situ effects in the brain^17^, which could explain our observation of cognitive impairment and brain abnormalities with mild changes in the periphery even long after the final episode of hepatotoxicity. Owing to the very limited knowledge of the metabolite’s neurological effects, our study is highly relevant because it sheds light on the cerebral and peripheral events that occur concomitantly in elderly hosts with history of APAP intoxication. Of note, our finding of cognitive impairment and increased gliosis in such subjects is also significant because of the confirmed roles for these phenotypes in other major aging-biased disorders, such as neurodegenerative diseases. In fact, a plethora of studies clearly indicate that neuroinflammation is important to the progressive brain dysfunction seen in demented persons, who exhibit an array of cognitive deficits including non-spatial memory impairment^72,73^. Our results are in agreement with these observations as markers for microglia (IBA1 and TMEM119) showed increased levels after chronic APAP administration. The fact that IBA1 was significantly increased in the cortex, and TMEM119 in the hippocampus, suggest that specific neuroinflammatory mechanisms are induced by APAP treatments. These will be explored as part of future studies.

Clinical and preclinical studies show that, in addition to cases of hepatic encephalopathy, liver dysfunction is implicated in increasing the susceptibility to Alzheimer’s disease, the leading cause of dementia, through yet elucidated mechanisms^74–76^. Our biochemical evaluation of livers from elderly subjects suggests that tissue function is compromised. Hence, this work gives insight into the cellular and molecular events that might underlie the speculative crosstalk between liver toxicity and dementia in persons at elevated risk for both pathological states.

Important questions remain regarding the involvement of numerous other known drivers of liver-driven brain irregularities. These include the following: (i) the hyperammonemia that inhibits key Krebs cycle enzymes to cause ATP depletion^77–80^; (ii) the increased cerebrovascular resistance that disrupts oxygen and nutrient supply in this tissue^81,82^; and, (iii) the accumulation of APAP in the brain^7,17^. Notably, while we did not query these biomarkers, it is worth considering that in cases of hepatic encephalopathy, such drivers of cerebral disruption occur concomitant with variably persistent and robust systemic inflammation. Also relevant, even when administered at concentrations considerably greater than ours (e.g., 1000 mg/Kg), APAP-induced hepatotoxicity in rodents can result in no changes to plasma ammonia levels or prothrombin time^83^. Thus, it is possible that our monthly APAP dosage of 300 mg/Kg, which appears to occur in an acute manner, did not result in hyperammonemia and or disruption of vascular integrity at the designated administration time points. Still, we did not assay associated biomarkers to conclusively interrogate this possibility. As noted earlier, our study data may also be explained by the cerebral accumulation of APAP since it is known to readily cross the BBB^7,8^. In the brain, this metabolite is linked to concentration-dependent protective and toxic effects (protective < 100 mg/Kg > toxic)^84^, albeit this appears to be influenced by the subject’s age at time of exposure and the frequency of treatments^17^. Indeed, Soares and colleagues report that four hours after a single excess dose of APAP, adult mice brains exhibit mitochondrial dysfunction and oxidative stress^85^. And yet, Viberg and colleagues show that at an adult life stage, the exact type and severity of cognitive deficits associated with a low APAP dose (30 mg/Kg) is dependent on whether mice had been pretreated with an identical concentration as neonates (10-days-old) or not^27^. The neurological contributions of APAP appear to be complex and age-dependent. This topic remains critically understudied under experimental conditions. Future such studies will benefit from more comprehensive evaluation of the brain congruent with the periphery, wherein the contributions of sex and other key parameters (treatment dose, concentration, and frequency) are queried at multiple life stages.

### Supplementary Information

Supplementary Legends.

Supplementary Figure S1.

Supplementary Figure S2.

Supplementary Table S1.

Supplementary Table S2.

## Data Availability

The data supporting the findings of this study are available on request from the corresponding author.

## References

[1] Ostapowicz G (2002). Results of a prospective study of acute liver failure at 17 tertiary care centers in the United States. Ann. Intern. Med..

[2] Gulmez SE (2015). Liver transplant associated with paracetamol overdose: Results from the seven-country SALT study. Br. J. Clin. Pharmacol..

[3] Chiew AL, Buckley NA (2021). Acetaminophen poisoning. Crit. Care Clin..

[4] Lee WM (2003). Acute liver failure in the United States. Semin. Liver Dis..

[5] Lee WM (2004). Acetaminophen and the U.S. Acute Liver Failure Study Group: Lowering the risks of hepatic failure. Hepatology.

[6] Clark R, Fisher JE, Sketris IS, Johnston GM (2012). Population prevalence of high dose paracetamol in dispensed paracetamol/opioid prescription combinations: An observational study. BMC Clin. Pharmacol..

[7] Courade JP (2001). Acetaminophen distribution in the rat central nervous system. Life Sci..

[8] Kumpulainen E (2007). Paracetamol (acetaminophen) penetrates readily into the cerebrospinal fluid of children after intravenous administration. Pediatrics.

[9] Esh CJ, Chrismas BCR, Mauger AR, Taylor L (2021). Pharmacological hypotheses: Is acetaminophen selective in its cyclooxygenase inhibition?. Pharmacol. Res. Perspect..

[10] Nelson SD (1990). Molecular mechanisms of the hepatotoxicity caused by acetaminophen. Semin. Liver Dis..

[11] Kane AE (2016). Acetaminophen hepatotoxicity in mice: Effect of age, frailty and exposure type. Exp. Gerontol..

[12] Ramachandran A, Jaeschke H (2019). Acetaminophen hepatotoxicity. Semin. Liver Dis..

[13] Bernal W, Wendon J (1998). The encephalopathy of acetaminophen induced acute liver failure is associated with cerebral endothelial activation. Crit. Care.

[14] Roth B, Woo O, Blanc P (1999). Early metabolic acidosis and coma after acetaminophen ingestion. Ann. Emerg. Med..

[15] Walls L, Baker CF, Sarkar S (2007). Acetaminophen-induced hepatic failure with encephalopathy in a newborn. J. Perinatol..

[16] Brusilow SW, Cooper AJL (2011). Encephalopathy in acute liver failure resulting from acetaminophen intoxication: New observations with potential therapy. Crit. Care Med..

[17] Ghanem CI, Pérez MJ, Manautou JE, Mottino AD (2016). Acetaminophen from liver to brain: New insights into drug pharmacological action and toxicity. Pharmacol. Res..

[18] McCormack JP, Brownlee S, Garber J, Devlin JW (2020). The endurance of medication overload: Rethinking the medication review process. JACCP J. Am. Coll. Clin. Pharm..

[19] Jansen PLM (2002). Liver disease in the elderly. Bailliere’s Best Pract. Res. Clinical Gastroenterol..

[20] Hou Y (2019). Ageing as a risk factor for neurodegenerative disease. Nat. Rev. Neurol..

[21] Corcoran GB, Mitchell JR, Vaishnav YN, Horning EC (1980). Evidence that acetaminophen and N-hydroxyacetaminophen form a common arylating intermediate, N-acetyl-p-benzoquinoneimine. Mol. Pharmacol..

[22] Dahlin DC, Miwa GT, Lu AYH, Nelson SD (1984). N-acetyl-p-benzoquinone imine: A cytochrome P-450-mediated oxidation product of acetaminophen. Proc. Natl. Acad. Sci. USA.

[23] Potter WZ, Thorgeirsson SS, Jollow DJ, Mitchell JR (1974). Acetaminophen induced hepatic necrosis. V. Correlation of hepatic necrosis, covalent binding and glutathione depletion in hamsters. Pharmacology.

[24] Speck RF, Schranz C, Lauterburg BH (1993). Prednisolone stimulates hepatic glutathione synthesis in mice. Protection by prednisolone against acetaminophen hepatotoxicity in vivo. J. Hepatol..

[25] Rogers LK, Moorthy B, Smith CV (1997). Acetaminophen binds to mouse hepatic and renal DNA at human therapeutic doses. Chem. Res. Toxicol..

[26] Posadas I, Santos P, Blanco A, Muñoz-Fernández M, Ceña V (2010). Acetaminophen induces apoptosis in rat cortical neurons. PLoS One.

[27] Viberg H, Eriksson P, Gordh T, Fredriksson A (2014). Paracetamol (acetaminophen) administration during neonatal brain development affects cognitive function and alters its analgesic and anxiolytic response in adult male mice. Toxicol. Sci..

[28] Lalert L (2020). Alterations in synaptic plasticity and oxidative stress following long-term paracetamol treatment in rat brain. Neurotox. Res..

[29] Yang J (2022). High-dose acetaminophen alters the integrity of the blood–brain barrier and leads to increased CNS uptake of codeine in rats. Pharmaceutics.

[30] Philippot G (2022). Paracetamol (acetaminophen) and its effect on the developing mouse brain. Front. Toxicol..

[31] Lalert L (2023). Long-term paracetamol treatment impairs cognitive function and brain-derived neurotrophic factor in adult rat brain. Sci. Pharm..

[32] Philippot G, Gordh T, Fredriksson A, Viberg H (2017). Adult neurobehavioral alterations in male and female mice following developmental exposure to paracetamol (acetaminophen): Characterization of a critical period. J. Appl. Toxicol..

[33] Vernacchio L, Kelly JP, Kaufman DW, Mitchell AA (2009). Medication use among children <12 years of age in the United States: Results from the Slone Survey. Pediatrics.

[34] Herndon CM, Dankenbring DM (2014). Patient perception and knowledge of acetaminophen in a large family medicine service. J. Pain Palliat. Care Pharmacother..

[35] Gyamlani GG, Parikh CR (2002). Acetaminophen toxicity: Suicidal vs accidental. Crit. Care.

[36] Thusius NJ, Romanowicz M, Bostwick JM (2019). Intentional or inadvertent acetaminophen overdose—How lethal it really is?. Psychosomatics.

[37] Federman D, Gruen JA, Merchant N (2021). Tylenol or acetaminophen: A recurrent fixed drug eruption perpetuated through the use of inconsistent drug terminology. BMJ Case Rep..

[38] Lane JE, Belson MG, Brown DK, Scheetz A (2002). Chronic acetaminophen toxicity: A case report and review of the literature. J. Emerg. Med..

[39] Heard K (2014). Toxicity from repeated doses of acetaminophen in children: Assessment of causes. Am. J. Ther..

[40] Mossanen JC, Tacke F (2015). Acetaminophen-induced acute liver injury in mice. Lab. Anim..

[41] Schomaker S (2013). Assessment of emerging biomarkers of liver injury in human subjects. Toxicol. Sci..

[42] Wang GX, Pan JY, Wang YJ, Huang TC, Li XF (2020). MiR-640 inhibition alleviates acute liver injury via regulating WNT signaling pathway and LRP1. Eur. Rev. Med. Pharmacol. Sci..

[43] Loft A (2021). Liver-fibrosis-activated transcriptional networks govern hepatocyte reprogramming and intra-hepatic communication. Cell Metab..

[44] Hamlin AN (2018). Low-density lipoprotein receptor-related protein-1 dysfunction synergizes with dietary cholesterol to accelerate steatohepatitis progression. J. Biol. Chem..

[45] Song Y, Liu J, Zhao K, Gao L, Zhao J (2021). Cholesterol-induced toxicity: An integrated view of the role of cholesterol in multiple diseases. Cell Metab..

[46] Finger CE, Moreno-Gonzalez I, Gutierrez A, Moruno-Manchon JF, McCullough LD (2022). Age-related immune alterations and cerebrovascular inflammation. Mol. Psychiatry.

[47] Moreno-Jiménez EP (2019). Adult hippocampal neurogenesis is abundant in neurologically healthy subjects and drops sharply in patients with Alzheimer’s disease. Nat. Med..

[48] Roe JM (2021). Asymmetric thinning of the cerebral cortex across the adult lifespan is accelerated in Alzheimer’s disease. Nat. Commun..

[49] McMillin M (2016). Bile acid signaling is involved in the neurological decline in a murine model of acute liver failure. Am. J. Pathol..

[50] McMillin M (2018). FXR-mediated cortical cholesterol accumulation contributes to the pathogenesis of type A hepatic encephalopathy. Cell. Mol. Gastroenterol. Hepatol..

[51] Liu Q (2010). Neuronal LRP1 knockout in adult mice leads to impaired brain lipid metabolism and progressive, age-dependent synapse loss and neurodegeneration. J. Neurosci..

[52] Lin JP, Mironova YA, Shrager P, Giger RJ (2017). LRP1 regulates peroxisome biogenesis and cholesterol homeostasis in oligodendrocytes and is required for proper CNS myelin development and repair. Elife.

[53] Cui W, Sun CM, Liu P (2013). Alterations of blood-brain barrier and associated factors in acute liver failure. Gastroenterol. Res. Pract..

[54] Dhanda S, Sandhir R (2018). Blood-brain barrier permeability is exacerbated in experimental model of hepatic encephalopathy via MMP-9 activation and downregulation of tight junction proteins. Mol. Neurobiol..

[55] Rossitto M (2019). In utero exposure to acetaminophen and ibuprofen leads to intergenerational accelerated reproductive aging in female mice. Commun. Biol..

[56] Mohar I (2014). Acetaminophen-induced liver damage in mice is associated with gender-specific adduction of peroxiredoxin-6. Redox Biol..

[57] Du K, Williams CD, McGill MR, Jaeschke H (2014). Lower susceptibility of female mice to acetaminophen hepatotoxicity: Role of mitochondrial glutathione, oxidant stress and c-jun N-terminal kinase. Toxicol. Appl. Pharmacol..

[58] Li J, Chiew AL, Isbister GK, Duffull SB (2021). Sulfate conjugation may be the key to hepatotoxicity in paracetamol overdose. Br. J. Clin. Pharmacol..

[59] Fisher ES, Curry SC (2019). Evaluation and treatment of acetaminophen toxicity. Advances in Pharmacology.

[60] Prescott LF (1979). Intravenous N-acetylcysteine: The treatment of choice for paracetamol poisoning. Br. Med. J..

[61] James LP, McCullough SS, Lamps LW, Hinson JA (2003). Effect of N-acetylcysteine on acetaminophen toxicity in mice: Relationship to reactive nitrogen and cytokine formation. Toxicol. Sci..

[62] Lauterburg BH, Corcoran GB, Mitchell JR (1983). Mechanism of action of N-acetylcysteine in the protection against the hepatotoxicity of acetaminophen in rats in vivo. J. Clin. Investig..

[63] Nakhaee S, Dastjerdi M, Roumi H, Mehrpour O, Farrokhfall K (2021). N-acetylcysteine dose-dependently improves the analgesic effect of acetaminophen on the rat hot plate test. BMC Pharmacol. Toxicol..

[64] De Gonzalo-Calvo D (2015). Circulating soluble low-density lipoprotein receptor-related protein 1 (sLRP1) concentration is associated with hypercholesterolemia: A new potential biomarker for atherosclerosis. Int. J. Cardiol..

[65] van Loo G, Bertrand MJM (2023). Death by TNF: A road to inflammation. Nat. Rev. Immunol..

[66] Chiu YC, Chu PW, Lin HC, Chen SK (2021). Accumulation of cholesterol suppresses oxidative phosphorylation and altered responses to inflammatory stimuli of macrophages. Biochem. Biophys. Rep..

[67] Deshmukh SS, Knierim JJ (2011). Representation of non-spatial and spatial information in the lateral entorhinal cortex. Front. Behav. Neurosci..

[68] Van Cauter T (2013). Distinct roles of medial and lateral entorhinal cortex in spatial cognition. Cereb. Cortex.

[69] Nikolakopoulou AM (2021). Endothelial LRP1 protects against neurodegeneration by blocking cyclophilin A. J. Exp. Med..

[70] Mach J (2014). The effect of aging on acetaminophen pharmacokinetics, toxicity and Nrf2 in fischer 344 rats. J. Gerontol. Ser. A Biol. Sci. Med. Sci..

[71] Liukas A (2011). Pharmacokinetics of intravenous paracetamol in elderly patients. Clin. Pharmacokinet..

[72] Ahmad MH, Fatima M, Mondal AC (2019). Influence of microglia and astrocyte activation in the neuroinflammatory pathogenesis of Alzheimer’s disease: Rational insights for the therapeutic approaches. J. Clin. Neurosci..

[73] Dhapola R (2021). Recent advances in molecular pathways and therapeutic implications targeting neuroinflammation for Alzheimer’s disease. Inflammopharmacology.

[74] Kim DG (2016). Non-alcoholic fatty liver disease induces signs of Alzheimer’s disease (AD) in wild-type mice and accelerates pathological signs of AD in an AD model. J. Neuroinflammation.

[75] Bosoi CR (2021). High-fat diet modulates hepatic amyloid β and cerebrosterol metabolism in the triple transgenic mouse model of Alzheimer’s disease. Hepatol. Commun..

[76] Giannisis A (2022). Brain integrity is altered by hepatic APOE ε4 in humanized-liver mice. Mol. Psychiatry.

[77] Sies H, Haeussinger D, Grosskopf M (1974). Mitochondrial nicotinamide nucleotide systems: Ammonium chloride responses and associated metabolic transitions in hemoglobin free perfused rat liver. Biol. Chem..

[78] Ong JP (2003). Correlation between ammonia levels and the severity of hepatic encephalopathy. Am. J. Med..

[79] Bernal W (2007). Arterial ammonia and clinical risk factors for encephalopathy and intracranial hypertension in acute liver failure. Hepatology.

[80] Marino RT, Sidlak AM (2022). Hyperammonemia in acetaminophen toxicity. Clin. Toxicol..

[81] Ponziani FR (2019). Minimal hepatic encephalopathy is associated with increased cerebral vascular resistance. A transcranial doppler ultrasound study. Sci. Rep..

[82] Caracuel L (2020). Hepatic encephalopathy-associated cerebral vasculopathy in acute-on-chronic liver Failure: Alterations on endothelial factor release and influence on cerebrovascular function. Front. Physiol..

[83] Isobe-harima Y (2008). A new hepatic encephalopathy model to monitor the change of neural amino acids and astrocytes with behaviour disorder. Liver Int..

[84] NazIroǧlu M, Cihangir Uǧuz A, Koçak A, Bal R (2009). Acetaminophen at different doses protects brain microsomal Ca2+-ATPase and the antioxidant redox system in rats. J. Membr. Biol..

[85] Da Silva MH (2012). Acute brain damage induced by acetaminophen in mice: Effect of diphenyl diselenide on oxidative stress and mitochondrial dysfunction. Neurotox. Res..

