# Supplementary material for: Elderly mice with history of acetaminophen intoxication display worsened cognitive impairment and persistent elevation of astrocyte and microglia burden
Source: Sci Rep. 2024 Jun 20;14:14205. doi: 10.1038/s41598-024-65185-z (PMC11190293; doi:10.1038/s41598-024-65185-z)
Supplement: Supplementary file 1 — Supplementary Legends. [file 41598_2024_65185_MOESM1_ESM.docx]

**SUPPLEMENTARY FIGURE LEGENDS**

**Supplementary Figure S1. ALT levels and survival of male and female mice repeatedly treated with APAP.** APAP-treated male mice exhibited significantly higher circulating levels of ALT and mortality compared to their female counterpart. Male and female mice sera collected each month since start of treatment was evaluated for ALT activity to query circulating levels of the liver injury biomarker (*n* = 8 and 6, for PBS and APAP, respectively). A graph of results is shown alongside a table outlining the 2-way repeated measures mixed effects model analysis, which are further detailed as Supplementary Table S2 online **(A)**. Survival curve analysis of experimental male and female subjects **(B)**.

**Supplementary Figure S2**. **Repetitive APAP treatment induces long-term increase in the microglia-specific marker TMEM119**. Elderly female mice displayed elevated microglial burden at one month since the final episode of APAP intoxication. Female mice were subjected to monthly intraperitoneal injection with saline vehicle (PBS) or APAP starting at 90 days of age until 420-days-old (*n* = 8 and 6, respectively). At 450 days of age, mice brains’ were isolated and submitted to immunofluorescence (IF) analysis for the TMEM119 protein. Representative images of IF staining of frozen brain sections from PBS- and APAP-treated female mice for TMEM119, a microglia-specific marker, in the cortex (**A**) and hippocampus (**C**) regions. Scale bars represent 100 µm. ImageJ analysis was used to quantify cortical (B) and hippocampal (**D**) levels of microglia. Student’s t-test. * *p* < 0.05.

**Supplementary Table S1. Details of 2-way repeated measures ANOVA followed by Tukey’s post hoc test for comparison of serum ALT activity between APAP- and PBS-treated females.**

**Supplementary Table S2. Details of 2-way repeated measures mixed effects model analysis followed by Tukey’s post hoc test for comparison of serum ALT activity between all male and female groups.**
