# Supplementary figures and images for: Elderly mice with history of acetaminophen intoxication display worsened cognitive impairment and persistent elevation of astrocyte and microglia burden
Source: Sci Rep. 2024 Jun 20;14:14205. doi: 10.1038/s41598-024-65185-z (PMC11190293; doi:10.1038/s41598-024-65185-z)

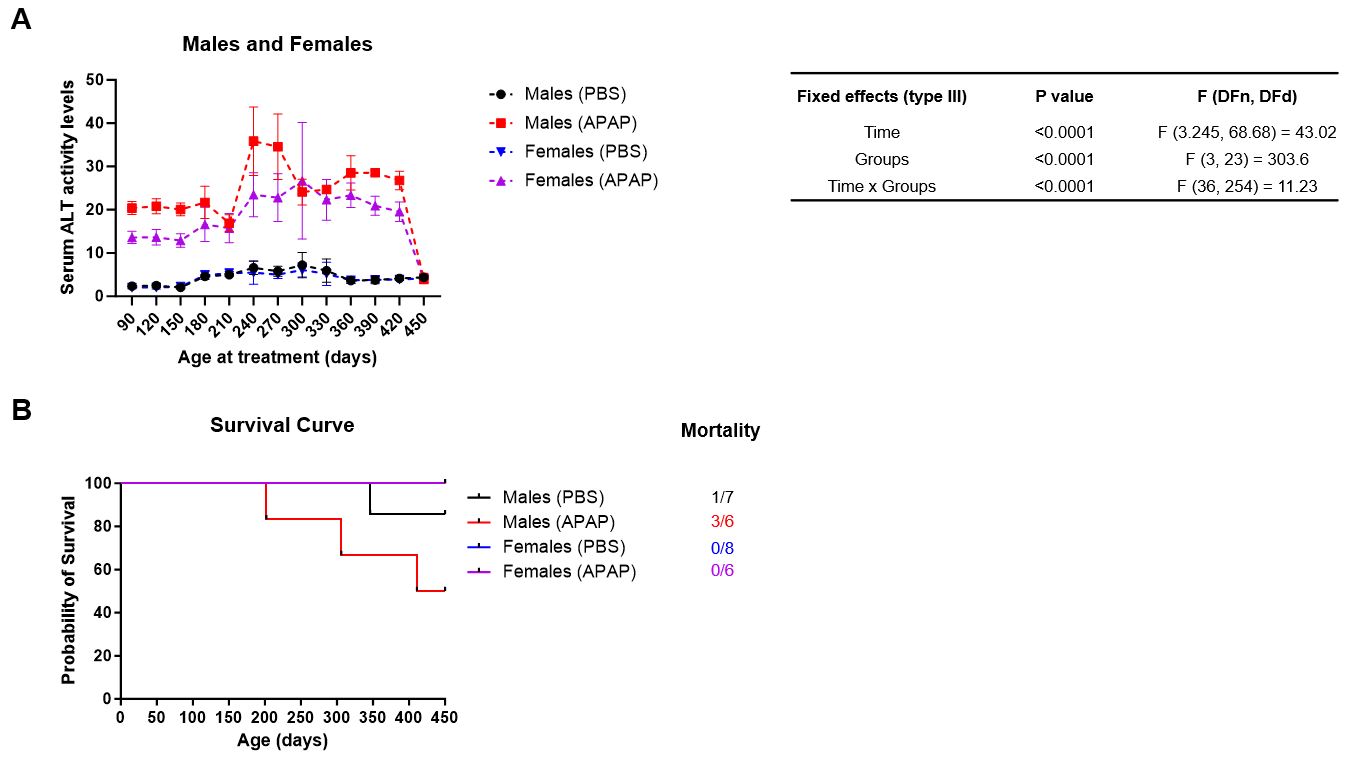

Supplement: Supplementary file 2 — Supplementary Figure S1. [file 41598_2024_65185_MOESM2_ESM.jpg]

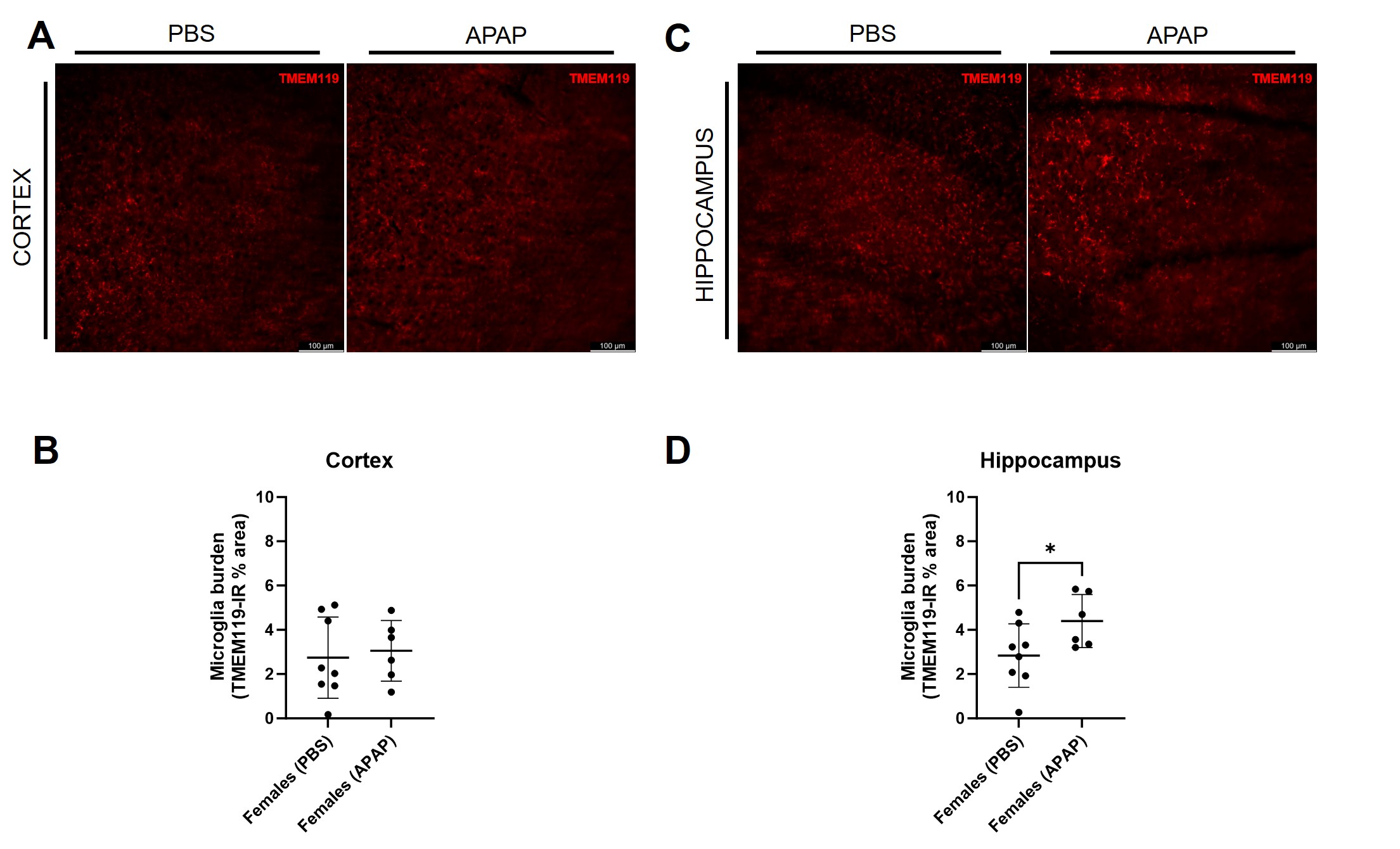

Supplement: Supplementary file 3 — Supplementary Figure S2. [file 41598_2024_65185_MOESM3_ESM.jpg]
